# Genomic and transcriptomic insights into the molecular responses of a biocrust-derived oleaginous microalga *Vischeria* sp. WL1 to nitrogen depletion and recovery

**DOI:** 10.1016/j.synbio.2025.06.004

**Published:** 2025-06-14

**Authors:** Xiaolan Rao, Wensheng Liang, Xin Jing, Chang Liu, Jiahong Li, Limei Liu, Xiang Gao

**Affiliations:** a State Key Laboratory of Biocatalysis and Enzyme Engineering, School of Life Sciences, Hubei University, Wuhan 430062, China; b College of Food Science and Engineering, Shaanxi University of Science and Technology, Xi'an 710021, China; c College of Bioengineering, Sichuan University of Science and Engineering, Yibin 644000, China

**Keywords:** Microalgae, Eustigmatophyte, Genome sequencing, Transcriptome analysis, Nitrogen depletion and recovery

## Abstract

Microalgae surviving in extreme conditions evolve tolerance mechanisms to diverse environmental stresses. Environmental nitrogen limitation or nitrogen starvation poses a significant threat to the growth of microalgae, but it is an effective factor in inducing lipid accumulation in many microalgal species. In this study, we report the genome sequence of a promising oil-producing strain, *Vischeria* sp. WL1, which is capable of resisting long-term nitrogen starvation and responds rapidly to nitrogen recovery. Its genome comprises 127 Mb of nuclear DNA and 17,392 protein-coding genes. Comparative genomic and transcriptomic analyses identified a group of genes involved in nitrate metabolism and lipid accumulation. Heterologous expression of three candidate genes in the model cyanobacterium *Synechocystis* sp. PCC 6803 demonstrated their effects on restoring the cellular chlorophyll upon nitrogen recovery. Our results provide novel insights into the molecular basis of metabolic shifts in response to nitrogen starvation and recovery in microalgae.

## 1 Introduction

Microalgae are typically single-celled photosynthetic eukaryotic organisms that thrive in diverse extreme environments such as saline waters and arid lands [1,2]. Their highly flexible metabolism enables microalgae to tolerate various environmental stresses, such as temperature fluctuations, salinity stress, and nitrogen deprivation. Due to their ease of growth, adaptability to stressful conditions, and ability to accumulate lipids, microalgae are globally recognized as a potential resource for alternative energy production [3,4].

Nitrogen is essential for the growth of microalgae as it constitutes basic cellular components such as proteins, amino acids, nucleic acids, and chlorophylls [5]. The growth rate, biochemical composition, and transcriptional profiles in response to long-term nitrogen depletion and subsequent recovery have been extensively studied in several microalgal species, including *Nannochloropsis gaditana* [[6], [7], [8]], *Nannochloropsis oceanica* [9,10], and *Chlamydomonas reinhardtii* [11]. Nitrogen deficiency disrupts photosynthetic capacity, significantly reduces chlorophyll content [12], and triggers a global decrease in protein expression [10]. However, nitrogen deficiency is also a key regulatory factor for enhancing the content of triacylglycerol [[13], [14], [15], [16]]. The induction of lipid biosynthesis relies not only on the upregulation of genes involved in fatty acid biosynthesis, but more crucially on the redirection of photosynthetic carbon partitioning toward triacylglycerol [[7], [8], [9]]. The molecular mechanisms underlying carbon flow direction under nitrogen limitation and subsequent nitrogen re-addition remain to be further explored, which is crucial for optimizing both lipid productivity and large-scale cultivation in the microalgae-based biofuel industry.

Many species in the Eustigmatophyceae class are considered promising strains due to their rapid growth and high accumulation of valuable compounds such as eicosapentaenoic acid and carotenoids [17]. Commercial applications of species from the genera *Nannochloropsis* and *Microchloropsis* were initially focused on [17,18]. Recently, certain members of the genus *Vischeria*, such as *Vischeria* sp. C74, *Vischeria* sp. H4302, and *V. stellata* SAG 33.83, have attracted growing attention [[19], [20], [21], [22]]. To date, genome assemblies have been reported for 11 strains across six species of *Nannochloropsis* and *Microchloropsis*, two strains of *Monodopsis*, and three strains of *Vischeria*; the completeness scores of these reported genome assemblies range from 59 % to 92 % [7,17,18,[21], [22], [23], [24], [25], [26], [27], [28], [29]]. Exploring the genomic information of new and promising microalgal species/strains is highly necessary. When integrated with transcriptome analysis, such exploration will lay a foundation for further investigation into their molecular responses and metabolic shifts in relation to nitrogen status and lipid accumulation.

Previously, we isolated a novel strain of Eustigmatophyceae*,* named *Vischeria* sp. WL1, from the arid steppe of north-western China [2]. This biocrust-derived strain is capable of accumulating high levels of C16:1 (∼50 %) and C20:5 (∼10 %) fatty acids and can be cultivated using salt lake water or organic matter-rich wastewater [2,30,31], and temperature-dependent two-stage cultivation [32,33]. Additionally, it can withstand long-term nitrogen starvation [34], exhibiting the potential for prolonged oil accumulation under nitrogen deprivation. These features suggest that it is a promising candidate for biofuel production applications.

In this study, a high-quality genomic assembly of *Vischeria* sp. WL1 and transcriptome profiling in response to nitrogen deficiency and recovery, were conducted. These studies will enhance the understanding of diversity within the Eustigmatophyceae class and molecular changes associated with lipid metabolism, thus facilitating better exploration of its applications in the future.

## 2 Materials and methods

### 2.1 Algal strain and cultivation condition

*Vischeria* sp. WL1 was previously isolated from a large biocrust on the eastern side of Helan Mountain in Yinchuan, Ningxia Hui Autonomous Region, China [2]. The strain has been preserved in the China Center for Type Culture Collection (CCTCC NO: M20211209). It was regularly cultivated in BG11 medium under continuous LED white illumination of 60 μmol photons m^−2^ s^−1^ at 25 °C.

### 2.2 Genome sequencing and assembly

The cell culture of *Vischeria* sp. WL1 was harvested by centrifugation at 2780×*g* for 5 min, and then subjected to genome sequencing by Bioyigene Biotechnology Co., Ltd. (Wuhan, China). In brief, the complete genome of *Vischeria* sp. WL1 was extracted using the Genomic Kit (QIAGEN, China). Qualified libraries were constructed using the Ligation Sequencing Kit (SQK-LSK109) and sequenced on the PromethION platform (Oxford Nanopore Technology). Basecalling was performed using Guppy v3.2.2 with a default threshold of 1 kb. NextDenovo v2.0 was employed to assemble the Nanopore reads, setting the genome size at 130 Mb. The assemblies were then error-corrected using Nanopore reads with Racon v1.4.10 over two iterations and polished with Illumina reads using Nextpolish v1.1.0 [35] over four iterations. The integrity of the genome assemblies was evaluated using BUSCO v4.0.3 [36] and CEGMA v2.5 [37].

### 2.3 Repeat sequence identification

Tandem repeats were identified using GMATA [38] and Tandem Repeats Finder [39]. Simple repeats within the genome were identified using MITE-hunter [40], LTR_finder [41], ltr_harvest [42], and RepeatModeler v1.0.11 [43], which were then integrated into a custom repeat database. All repeat classes were annotated and masked from the assembled genome using RepeatMasker v4.0.9 (http://www.repeatmasker.org).

### 2.4 Gene model predication and annotation

Gene models were predicted via three approaches: transcript alignment using PASA v2.3.3 [44], homology alignment using GeMoMa v1.6.1 [45], and *de novo* prediction using Augustus v3.3.1 [46]. The results were integrated using EVM [47] and filtered with TransposonPSI (http://transposonpsi.sourceforge.net) to remove transposable elements. Non-coding RNA was predicted using tRNAscan-SE v2.0.5 [48] and RNAmmer v1.2 [49], and searched against the non-coding RNA database Rfam [50] with Infernal v1.1.2 [51]. For protein-coding sequences, gene function was annotated by searching against NR (Non-Redundant Protein Database), KEGG, GO, KEGG, and SwissProt using Blastp v2.7.1 with a threshold of 1e-5. InterProScan v5.32–71.0 [52] was used to predict GO functions.

### 2.5 Phylogenetic analysis

Twelve nuclear genomes were selected for evolutionary analysis, including *Vischeria* sp. WL1, *Vischeria* sp. CAUP H4302, *Vischeria stellata* SAG 33.83, *Vischeria* sp. C74, *Monodopsis* sp. C141, *Monodopsis* sp. C73, *Nannochloropsis salina* CCMP1776, *Nannochloropsis gaditana* CCMP1894, *Nannochloropsis gaditana* B-31, *Nannochloropsis oceanica* CCMP1779, *Thalassiosira oceanica* CCMP1005, and *Thalassiosira pseudonana* CCMP 1335. The *Vischeria* sp. WL1 genome was assembled in this study, while other genomes were obtained from CNGBdb (https://db.cngb.org/) or PhycoCosm (https://phycocosm.jgi.doe.gov) [53]. Orthologous groups among the twelve algal species were generated using OrthoFinder v2.5.5 [54]. The phylogenetic tree was created using OrthoFinder v2.5.5 with default settings. An 18S rDNA sequence obtained from *Vischeria* sp. WL1 and thirty-nine additional 18S rDNA sequences downloaded from NCBI database were aligned using ClustalW [55]. The maximum likelihood (ML) tree with 1000 bootstrap replicates was constructed using MEGA 11 [56]. Individual gene family trees were inferred using FastTree (-lg -gamma -spr 4) (http://meta.microbesonline.org/fasttree/) and visualized with MEGA 11.

### 2.6 Sample preparation for transcriptomic analysis

Prior to nitrogen-starvation treatment, logarithmic-phase algal cells grown in BG11 medium were harvested by centrifugation (2780×*g*, 5 min). The resulting pellets were fully rinsed with BG11_0_ medium. Subsequently, the rinsed cells were evenly divided into four Erlenmeyer flasks, each containing 100 mL of BG11_0_ medium, for nitrogen starvation treatment. Samples were collected at 0, 3, 9, and 24 h after nitrogen deprivation (numbered as D0, D3, D9, and D24, respectively). Each time, 20 mL of the culture was taken and immediately placed on ice. After centrifugation at 4 °C, the pellets were quickly frozen in liquid nitrogen for use.

Prior to nitrogen recovery treatment, algal cells were cultivated in 100 mL of BG11 medium for two months, then collected and rinsed with BG11_0_ medium. The rinsed cells were subcultivated in 100 mL of BG11_0_ medium for two months, and after that, the cells were transferred to fresh BG11_0_ medium for the next two months of cultivation. Subsequently, the cells were collected and evenly divided into four Erlenmeyer flasks, each containing 100 mL of BG11_0_ medium. Each flask was then supplemented with 1 mL of 100 mM NaNO_3_ stock solution (resulting in a final concentration of 1 mM) for nitrogen recovery treatment. Samples were collected at 0, 1, 3, 6, and 24 h after nitrogen addition (numbered as N0, N1, N3, N6 and N24, respectively). Each time, 15 mL of the culture was taken and immediately placed on ice. After centrifugation, the pellets were similarly frozen for use.

### 2.7 RNA sequencing and data analysis

Totally, 16 samples from the nitrogen-starvation group and 20 samples from the nitrogen-recovery group were sent to Shanghai Applied Protein Technology Co., Ltd. (China) for RNA sequencing. Thus, thirty-six RNA-seq libraries were constructed according to the Illumina's standard protocol. The qualified libraries were sequenced on the Illumina HiSeq 2000 platform. 150 bp paired-end reads were generated and trimmed using fastp v0.23.3 [57]. The obtained clean reads were mapped to the *Vischeria* sp. WL1 reference genome using TopHat v2.1.1 [58] with default parameters. Gene expression was normalized and quantified using the FPKM method. Differentially expressed genes (DEGs) were identified using Cufflinks v2.2.1 [59] with an adjusted *P*-value cutoff of <0.05.

### 2.8 Validation of gene function *via* transgenic *Synechocystis* sp. PCC 6803

The starting plasmid for construction was pUC57-simple (conferring ampicillin resistance). The left and right 500-bp sequences flanking the neutral site *slr0168* were used for homologous recombination in *Synechocystis* sp. PCC 6803. A cassette fragment, which contains the kanamycin resistance cassette, *psbA2* promoter, ribosome binding site (RBS), restriction-enzyme cutting sites, and B0050 terminator, was designed for insertion between the two sequences. These nucleic acid fragments were synthesized by GeneScript Biotech Corporation, China (https://www.genscript.com/), ultimately resulting in pUC57-6803-frame vector. Next, three genes, evm.model.contig000115.7 (*R7*), evm.model.contig000029.171 (*N171*), and evm.model.contig000024.186 (*N186*), were synthesized and separately subcloned into the pUC57-6803-frame using *Spe*I and *Xho*I restriction sites under the control of the *psbA2* promoter. The sequences for pUC57-6803-frame vector and three genes are summarized in Supplementary Data Set S1. Transformation procedures and transformant selection were carried out as previously described [60]. Positive transformants were identified by PCR, and homozygous transformants were subjected to growth testing. Cells were cultivated under an LED light intensity of 30 μmol photons m^−2^ s^−1^ at 30 °C in a shaker (130 rpm). The culture media used were BG11_0_ medium or BG11_0_ medium supplemented with 1 mM NaNO_3_. Growth (measured as OD_750_) and pigment contents (chlorophyll *a* and carotenoids) were determined as previously described [2]. The cell density was calibrated such that a density of 1 × 10^8^ cells mL^−1^ approximately corresponded to 0.25 OD_750_.

## 3 Results and discussion

### 3.1 Nitrogen availability-responsive features in *Vischeria* sp. WL1

**Fig. 1:**
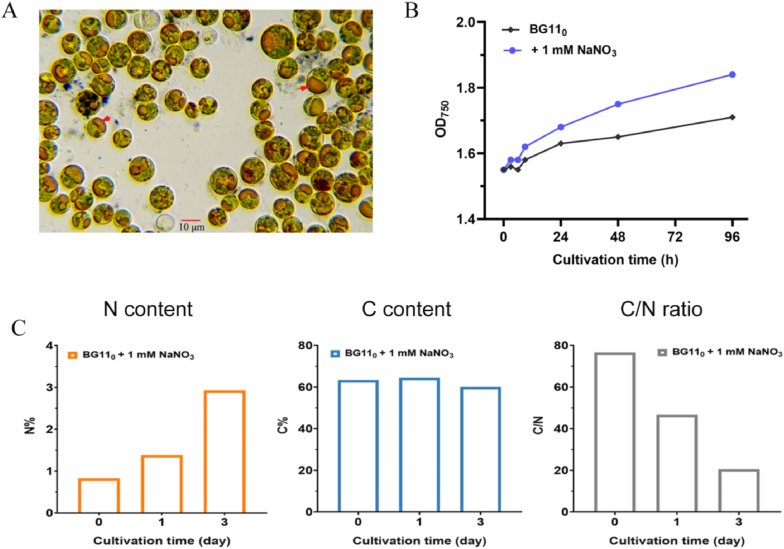
Growth characteristics of *Vischeria* sp. WL1. (A) Oil droplets in *Vischeria* sp. WL1 cells after cultivation in ammonium acetate-supplemented BG11 medium. Red arrows point to oil droplets. Cells were subjected to staining by Sudan black (partially stained). (B) Growth (measured by OD_750_) of long-term nitrogen-starved *Vischeria* sp. WL1 in fresh BG11_0_ medium with or without 1 mM NaNO_3_ supplementation. (C) N and C contents, and C/N ratios of cells after three days of culturing nitrogen-starved cells in fresh BG11_0_ medium supplemented with 1 mM NaNO_3_. The nitrogen starvation treatment of cells was performed as previously described [34].

*Vischeria* sp. WL1 is an oil-producing strain of Eustigmatophyceae that was isolated from a biocrust. Like other members of Eustigmatophyceae such as *Nannochloropsis* sp., it exhibits excellent oil-accumulation capability (more than 50 % oil content) [2]. As shown in Fig. 1A, oil droplets could be obviously observed in the cells of *Vischeria* sp. WL1. Nitrogen deficiency is widely used for inducing oil production in microalgae [61,62]. Similarly, a reduced nitrogen level could induce oil accumulation in *Vischeria* sp. WL1 [2]. The biomass increase and nitrogen consumption rates of this strain under different initial nitrogen levels are shown in Supplemental Fig. S1. However, most interestingly, this strain showed remarkable adaptation to long-term nitrogen starvation (up to 180 days) without being rested or dying [34]. A similar study was from *N. gaditana*, yet it was cultivated for 150 days under a semi-continuous low-nitrogen condition [8].

When long-term nitrogen-starved cells were transferred to BG11_0_ medium supplemented with 1 mM NaNO_3_, they exhibited rapid growth recovery (Fig. 1B). The nitrogen starvation treatment was conducted as previously described [34]. Moreover, the cellular nitrogen (N) content also rapidly increased, whereas the carbon/nitrogen (C/N) ratio decreased rapidly (Fig. 1C). By day 3, the N content had recovered from 0.83 % to 2.93 %, and the C/N ratio had dropped from 76.6 to 20.5. When the nitrogen-starved cells were transferred to BG11_0_ medium supplemented with 18 mM NaNO_3_, these changes occurred even more rapidly (Supplementary Fig. S2). Collectively, these results indicate that *Vischeria* sp. WL1 exhibits great resilience to ambient nitrogen status.

In the following study, we sequenced its genome and conducted transcriptomic analysis of its early stress responses to nitrogen depletion and recovery to better understand its genetic background and nitrogen-related metabolic features.

### 3.2 High-quality assemblies of the *Vischeria* sp. WL1 genome

To obtain the genetic information of *Vischeria* sp. WL1, we sequenced its genome using Oxford Nanopore long reads. A total of 14.8 Gbp clean reads (N50 length of 21.7 kbp, approximately 107-fold coverage of the genome) were generated on the PromethION System. The genome was assembled, corrected, and polished using both Oxford Nanopore long reads and Illumina short reads (approximately 120-fold coverage of the genome), resulting in a 127 Mbp genome size with 200 contigs. The N50 and maximum contig length were 1080 Kbp and 3319 Kbp, respectively. The completeness of the genome was evaluated using the Benchmarking Universal Single-Copy Ortholog (BUSCO) assessment and the Core Eukaryotic Genes Mapping Approach (CEGMA). The results showed 84 of 100 (84 %) and 216 of 248 (87 %) complete BUSCOs and complete core eukaryotic genes, respectively. Compared to the average completeness score of 83 % in the 17 published genome assemblies of the Eustigmatophyceae class [22], our results suggest that a high-quality genome for *Vischeria* sp. WL1 was achieved (Table 1, Tables S1–2).

**Table 1:** General features of *Vischeria* sp. WL1 genome.

**Genome statistics**	
Assembled genome size (Mbp)	127
Sequencing depth	X90
No. Of contigs	200
Contig N50 (kbp)	1080
Length of maximum contig (kbp)	3319
BUSCO, genome assembly	84 %
CEGMA, genome assembly	87 %
**Gene statistics**	
No. Of protein coding genes	17,392
Average transcript length (bp)	3565
Average CDS length (bp)	1550
Average exons number per gene	8
Average exon length (bp)	179
Average intron length (bp)	263
BUSCO, gene prediction	97 %
Percentage of annotated genes	68.18 %
**Statistics for repeat sequences**	
Length percentage of repeat sequence	47.16 %
Length percentage of non-coding genes	0.06 %

A total of 17,392 non-redundant protein-coding genes were identified using gene modeling, homology searching, and RNA-seq data (Table S3). The average lengths of these transcripts and coding sequences (CDS) were 3565 bp and 1550 bp, respectively. Each gene contained an average of 8 exons, with average exon and intron lengths of 179 bp and 263 bp, respectively. The predicted gene models achieved a near-complete BUSCO score (97 %). These 17,392 genes were functionally annotated based on homology searches against five reference databases (the non-redundant (NR) NCBI database, GO, KEGG, KOG, and SwissProt). Among them, 68.2 % of the genes were found in at least one of these five reference databases (Table 1 and Table S4). *Vischeria* sp. WL1 transcripts showed the highest homology to transcripts from *N. gaditana* (33.4 %) and *N. salina* (31.1 %) in the NR database, consistent with the close phylogenetic relationship between *Vischeria* and *Nannochloropsis* + *Microchloropsis*. Additionally, 265 non-coding RNAs were predicted in the genome, occupying 0.06 % of the genome. Repeated sequences accounted for 47.16 % of the genomic content, with transposable elements (TEs) being the major component at 40.12 % (Table 1, Tables S5–7).

### 3.3 Comparative analysis of genome assemblies in eustigmatophyte algae

**Fig. 2:**
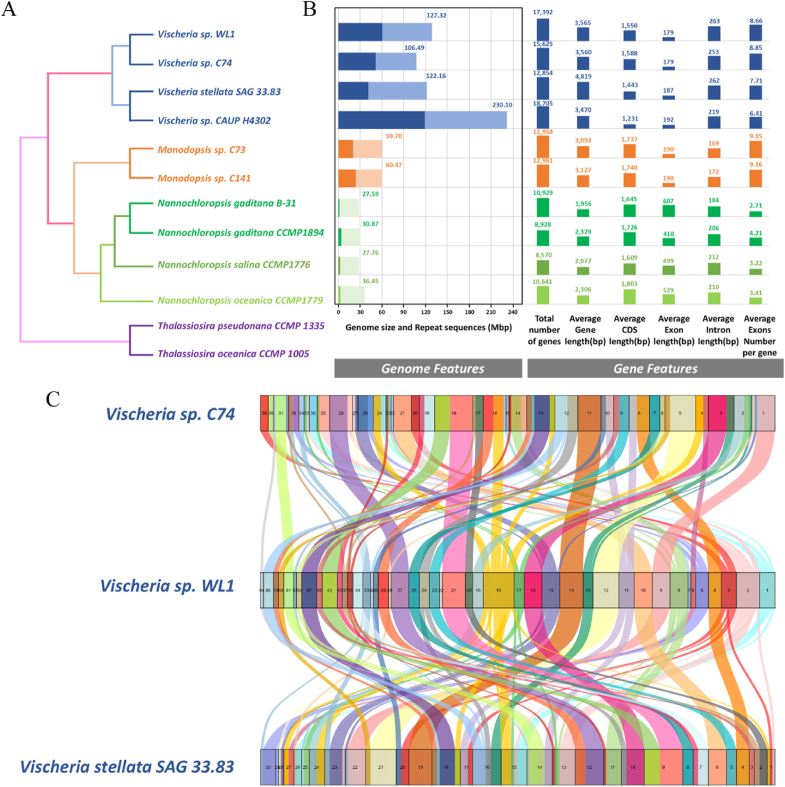
Genomic evolution and structural variation in Eustigmatophyceae. (A) Phylogenetic relationship of *Vischeria* sp. WL1 and its relatives inferred from nuclear genome. (B) Genomic characteristics of Eustigmatophyceae. (C) Syntenic orthologous blocks among *Vischeria* sp. WL1, *Vischeria* sp. C74, and *V. stellata* SAG 33.83.

To determine the phylogenetic position of *Vischeria* sp. WL1 within microalgae, we constructed phylogenetic trees using nuclear genome data and the 18S ribosomal RNA gene (18S rDNA). The genome of *Vischeria* sp. WL1 was compared with eleven other genomes, including three from the group *Vischeria*, two from *Monodopsis*, and four from *Nannochloropsis*, along with two from the family *Bacillariophyta* as an outgroup (Fig. 2A). A Maximum Likelihood phylogenetic tree was generated using 18S rDNA sequences representing *Vischeria*, *Monodopsis*, *Nannochloropsis*, *Microchloropsis*, *Neomonodaceae*, and *Goniochloridales* (Supplementary Fig. S3). The results indicated that *Vischeria* sp. WL1 belongs to the family Eustigmataceae, and is closely related to *Monodopsis* and the clade comprising *Nannochloropsis* and *Microchloropsis* within the family *Monodopsidaceae*. The closest relationship was observed between *Vischeria* sp. WL1 and *Vischeria* sp. C74, followed by *Vischeria* sp. H4302 and *V. stellata* SAG 33.83 (Fig. 2A).

The genomic features of Eustigmatophyte algae vary significantly. Four *Vischeria* strains exhibit extremely large genome sizes (106.5–224.9 Mb) with high proportions of repeated sequences (34.8–51.8 %). In contrast, two *Monodopsis* strains have moderate genome sizes (59.7–60.5 Mb) with 33.2–39.4 % repeated sequences, while the *Nannochloropsis* + *Microchloropsis* group shows small genome sizes (26.9–35.5 Mb) with low proportions of repeated sequences (1.9–8.7 %) (Fig. 2B). Moreover, the general gene characteristics—including gene number, gene length, exon number, and average lengths of exons and introns—are similar across all four *Vischeria* strains. However, these characteristics differ from those observed in the reference genomes of the *Monodopsis* and *Nannochloropsis* + *Microchloropsis* clades (Fig. 2B), suggesting that genome evolution in the *Vischeria* lineage may have diverged from other taxa.

To further explore the interspecific relationship within the genus *Vischeria*, we identified homologous genes between *Vischeria* sp. WL1 and the three other *Vischeria* strains. 85.6 % of genes in *Vischeria* sp. WL1 were found to have homologs in at least one other *Vischeria* strain. Additionally, *Vischeria* sp. WL1 shared 65.5 %, 57.8 %, and 57.3 % collinear genes with *Vischeria* sp. C74, *V. stellata* SAG 33.83, and *Vischeria* sp. H4302, respectively (Fig. 2C). The syntenic orthologous blocks suggest that *Vischeria* strains share a high level of evolutionary conservation at the whole-genome scale but evolve individually.

### 3.4 The transcriptional reprogramming under nitrogen depletion and recovery

**Fig. 3:**
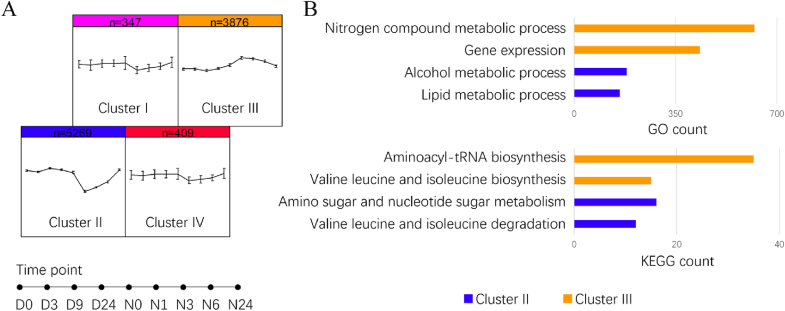
Overview of gene expression in response to nitrogen depletion and recovery. (A) Cluster analysis of DEGs using the SOM method. (B) Selected GO and KEGG pathway enrichment analyses of genes in expression cluster II and cluster III.

To assess the global changes in gene expression associated with the early stages of nitrogen depletion and recovery, we further conducted transcriptomic analysis. During nitrogen depletion for 24 h, the growth of *Vischeria* sp. WL1 was not significantly affected, whereas it was rapidly enhanced following nitrogen addition (Fig. 1B). RNA-Seq analysis was performed on *Vischeria* sp. WL1 cultures at 0, 3, 9, and 24 h following nitrogen deprivation and at 0, 1, 3, 6, and 24 h following nitrogen addition (Table S8). After filtering out genes with low expression levels, a self-organizing map (SOM) was applied to cluster the DEGs into four groups (Fig. 3A). Among these, cluster II represents genes with transcript levels up-regulated at 3 h in response to nitrogen deprivation and rapidly down-regulated at 3 h after nitrogen recovery. In contrast, cluster III represents genes with transcript levels that gradually decreased after nitrogen deprivation and increased after nitrogen replenishment. Furthermore, GO and KEGG analyses were used to compare the biological functions of genes between Cluster II and Cluster III (Fig. 3B and Table S9). Genes involved in lipid metabolic processes, alcohol metabolic processes, carbon fixation in photosynthetic organisms, and valine, leucine, and isoleucine degradation were significantly enriched in Cluster II. Conversely, genes involved in nitrogen compound metabolic processes and valine, leucine, and isoleucine biosynthesis were preferentially enriched in Cluster III. This suggests an intracellular redistribution of carbon and nitrogen flux. In general, our transcriptomic analysis reveals the metabolic trends associated with lipid and nitrogen metabolism during the early stages of nitrogen deficiency or recovery, providing molecular-level insights into short-term stress responses.

### 3.5 Metabolic pathway changes under nitrogen depletion and recovery

In the following section, we applied comparative genome and transcriptome analyses to investigate the underlying molecular mechanisms in response to nitrogen deprivation and recovery. We identified all genes associated with nitrogen metabolism, amino acid metabolic pathways, fatty acid synthesis, and triacylglycerol synthesis in ten genomes of the Eustigmataceae family. The gene expression profiles of *Vischeria* sp. WL1 in this study were compared with those of *Vischeria* sp. H4302 under nitrogen-limiting conditions [22].

#### 3.5.1 Genomic basis of low-nitrogen adaptation in *Vischeria* sp. WL1

**Fig. 4:**
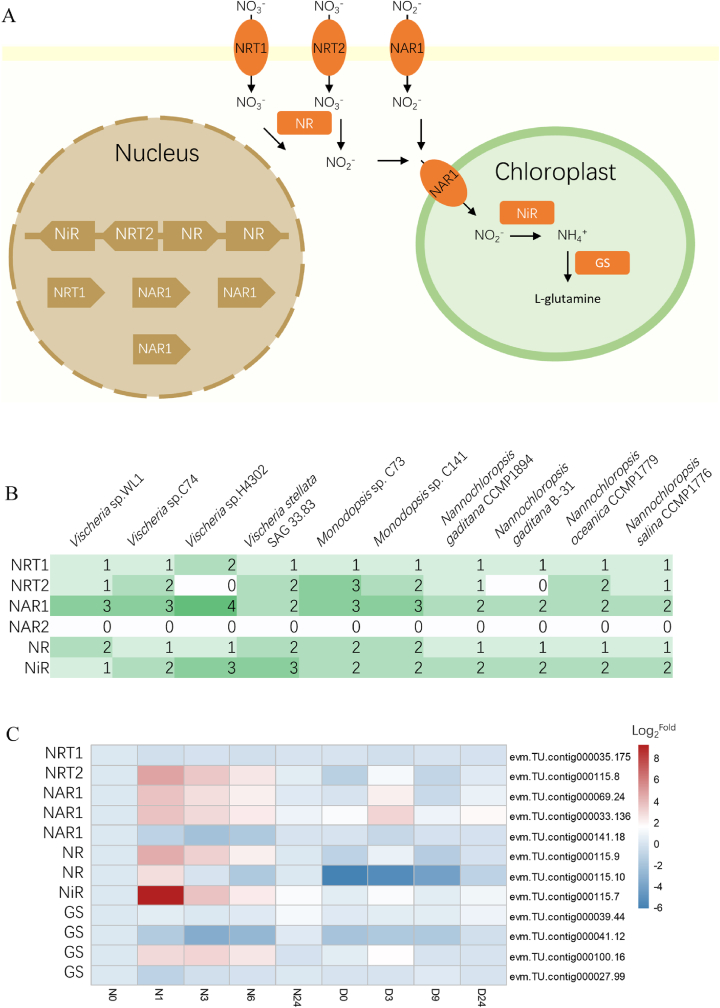
Evolution and expression of genes involved in the nitrogen metabolism pathway. (A) Simplified overview of the nitrate assimilation pathway in *Vischeria* sp. WL1. (B) Nitrate assimilatory proteins in Eustigmatophyceae microalgae. The color of rectangle represents the gene number in each row. (C) RNA expression of nitrogen metabolism enzymes in *Vischeria* sp. WL1. N0, N1, N3, N6, and N24 denote the time points (hours) following nitrogen addition; D0, D3, D9, and D24 denote the time points (hours) following nitrogen deprivation. The expression level was normalized to that at N0. NRT, nitrate/nitrite transporter; NAR, nitrate transporter component; NR, nitrate reductase; NiR, nitrite reductase; GS, glutamine synthetase.

In photosynthetic eukaryotes, the process of nitrate assimilation from nitrate to amino acids proceeds as follows [63]: first, nitrate is transported into the cell by nitrate transporters and subsequently reduced to nitrite by nitrate reductase (NR); nitrite is then transported into the chloroplast and converted to ammonium by nitrite reductase (NiR); finally, the resulting ammonium is assimilated into amino acids via the glutamine synthetase/glutamate synthase (GS/GOGAT) cycle (Fig. 4A).

##### 3.5.1.1 Nitrate transport and nitrite transport

NRT1, NRT2, and NAR1 have been well-characterized as nitrate/nitrite transporters in plants [63]. We identified genes homologous to *Chlamydomonas reinhardtii* NRT1, NRT2, and NAR1 in the ten Eustigmatophyceae genomes (Fig. 4B and Table S10), indicating a broad presence of these transporters in microalgae. NAR2 proteins interact with NRT2 to form a functional two-component system, commonly observed in Chlorophyte algae and plants [63,64]. However, homologs to NAR2 are absent in all ten Eustigmatophyceae genomes, suggesting that the NAR2 component might not be essential for NRT2 functionality.

##### 3.5.1.2 Nitrate reductase and nitrite reductase

In eukaryotes, the enzymes nitrate reductase (NR) and nitrite reductase (NiR) catalyze the reduction of nitrate to nitrite in the cytoplasm and the reduction of nitrite to ammonium in chloroplasts, respectively. Typically, a single NR gene is present in algal genomes [63,64]. We identified two copies of the NR gene in *Vischeria* sp. WL1, *V. stellata* SAG 33.83, and two *Monodopsis* species (Fig. 4B and Supplementary Fig. S4).

The expression levels of genes encoding nitrate transporters, nitrite transporters, nitrate reductase, and nitrite reductase were significantly regulated by nitrate availability in *Vischeria* sp. WL1 (Fig. 4C and Table S11), as observed in *Vischeria* sp. H4302 (Table S12) [22]. Notably, the expression of NRT2, NR, and NiR genes was maintained at high levels and was significantly regulated under nitrogen depletion in *Vischeria* sp. WL1. These four nitrate assimilation genes (NRT2, NiR, and two NR paralogs) form a complete cluster in the genome of *Vischeria* sp. WL1 (Fig. 4A), but not in *Vischeria* sp. H4302. The expression of these four genes was highly correlated (Pearson correlation coefficients of 0.92–0.99), indicating coordinated regulation. The clustering of nitrate assimilation genes may represent an adaptive strategy for nitrogen acquisition in *Vischeria* sp. WL1.

#### 3.5.2 Gene expression changes associated with lipid accumulation

Triacylglycerols (TAGs) serve as long-term energy storage molecules in cells and are predominantly produced by microalgae [65]. The biosynthesis of TAGs involves two main steps: fatty acid synthesis and glycerolipid synthesis.

##### 3.5.2.1 Fatty acid synthesis

**Fig. 5:**
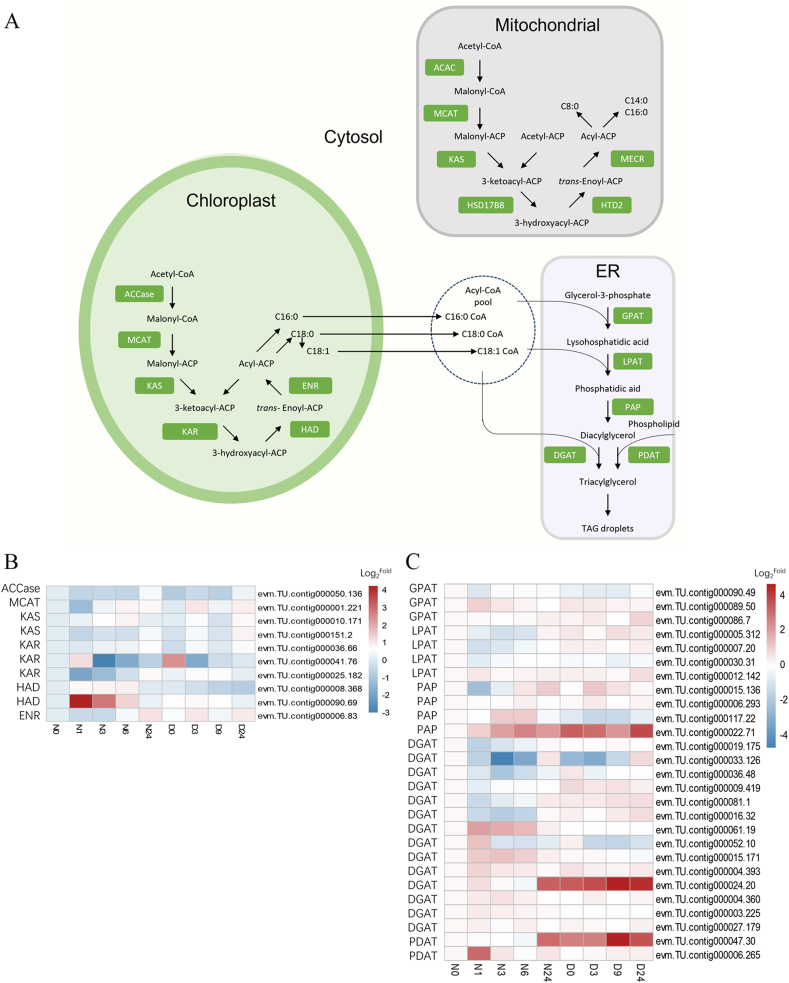
Expression of genes involved in the lipid biosynthetic pathway. (A) Overview of fatty acid and glycerolipid synthesis. (B) RNA expression of fatty acid synthesis-associated genes in *Vischeria* sp. WL1. (C) RNA expression of triacylglycerol synthesis-associated genes in *Vischeria* sp. WL1. N0, N1, N3, N6, and N24 denote the time points (hours) following nitrogen addition; D0, D3, D9, and D24 denote the time points (hour) following nitrogen deprivation. The expression level was normalized to that at N0. ER, endoplasmic reticulum; TAG, triacylglycerol; ACAC, acetyl-CoA carboxylase; MCAT, malonyl-CoA-acyl carrier protein transacylase; KAS, 3-ketoacyl-ACP synthase; HSD17B8, hydroxysteroid 17-beta dehydrogenase 8; HTD2, Hydroxyacyl-thioester dehydratase type 2; MECR, mitochondrial *trans*-2-enoyl-CoA reductase. ACCase, acetyl-CoA carboxylase; KAR, 3-ketoacyl-ACP reductase; HAD, hydroxyacyl-CoA dehydrogenase; ENR, enoyl-acyl carrier protein reductase; GPAT, glycerol-3-phosphate acyltransferase; LPAT, 1-acyl-*sn*-glycerol-3-phosphate acyltransferase; PAP, phosphatidic acid phosphatase; DGAT, acyl-CoA:diacylglcerol acyltransferase; PDAT, phospholipid:diacylglcerol acyltransferase.

In plants and algae, *de novo* fatty acid biosynthesis occurs in the plastids [66]. The precursor acetyl-CoA is ultimately converted into fatty acids through a series of biochemical reactions (Fig. 5A). The enzymes involved include acetyl-CoA carboxylase (ACCase), malonyl-CoA-acyl carrier protein transacylase (MCAT), β-ketoacyl-ACP synthase (KAS), β-ketoacyl-ACP reductase (KAR), β-hydroxyl-ACP dehydratase (HAD), and enoyl-ACP reductase (ENR) [66]. A high degree of correlation was observed in the expression of ACCase, MCAT, KAS, KAR, and ENR (Fig. 5B and Table S13), suggesting co-regulation of these enzymes in response to nitrogen deprivation and recovery. An exception is HAD, which was highly expressed 1 h after nitrogen addition, indicating a distinct rapid response of nitrogen-starved cells to nitrogen repletion.

In addition to fatty acid synthesis in plastids, we identified a group of genes encoding enzymes involved in the mitochondrial fatty acid pathway (Fig. 5B and Table S13). This finding is consistent with observations in *Vischeria* sp. H4302 and *V. stella* SAG 33.83, suggesting the occurrence of mitochondrial fatty acid synthesis in the genus *Vischeria*.

##### 3.5.2.2 Triacylglycerol synthesis

The products of fatty acid synthesis in plastids are converted to acyl-CoAs by long-chain acyl-CoA synthetases (LACSs) in the chloroplast envelope. These acyl-CoAs are then transported to the endoplasmic reticulum (ER) and incorporated into TAGs [67]. Two acyl-CoA-dependent acyltransferases (GPAT and LPAT) and phosphatidic acid phosphatase (PAP) are involved in the formation of diacylglycerol. TAGs can be synthesized from diacylglycerol by either DGAT (via the conventional Kennedy pathway) or PDAT [67].

The expression levels of genes encoding GPAT, LPAT, and PAP remained relatively stable under both nitrogen deprivation and repletion conditions in *Vischeria* sp. WL1 (Fig. 5C and Table S13). However, the expression of DGAT/PDAT genes showed much more fluctuations (Fig. 5C and Table S13). Given that different DGAT/PDAT classes contribute to the accumulation of specific fatty acid types in TAGs [67], these DGAT/PDAT genes may serve as candidates for regulating the fatty acid composition of TAGs in *Vischeria* sp. WL1.

In previous studies of *N. gaditana* [7] and *Vischeria* sp. H4302 [22], most genes involved in fatty acid and triacylglycerol synthesis did not exhibit differential expression under nitrogen deprivation. However, those RNA-seq analyses were performed at least two days after nitrogen deprivation. In the present study of *Vischeria* sp. WL1, we observed significant differential expression of a subset of genes in the lipid biosynthesis pathway within 24 h of nitrogen depletion. This suggests that specific transcriptional changes are rapidly regulated in the short term following nitrogen deprivation.

### 3.6 Experimental validation of genes associated with nitrate assimilation

**Fig. 6:**
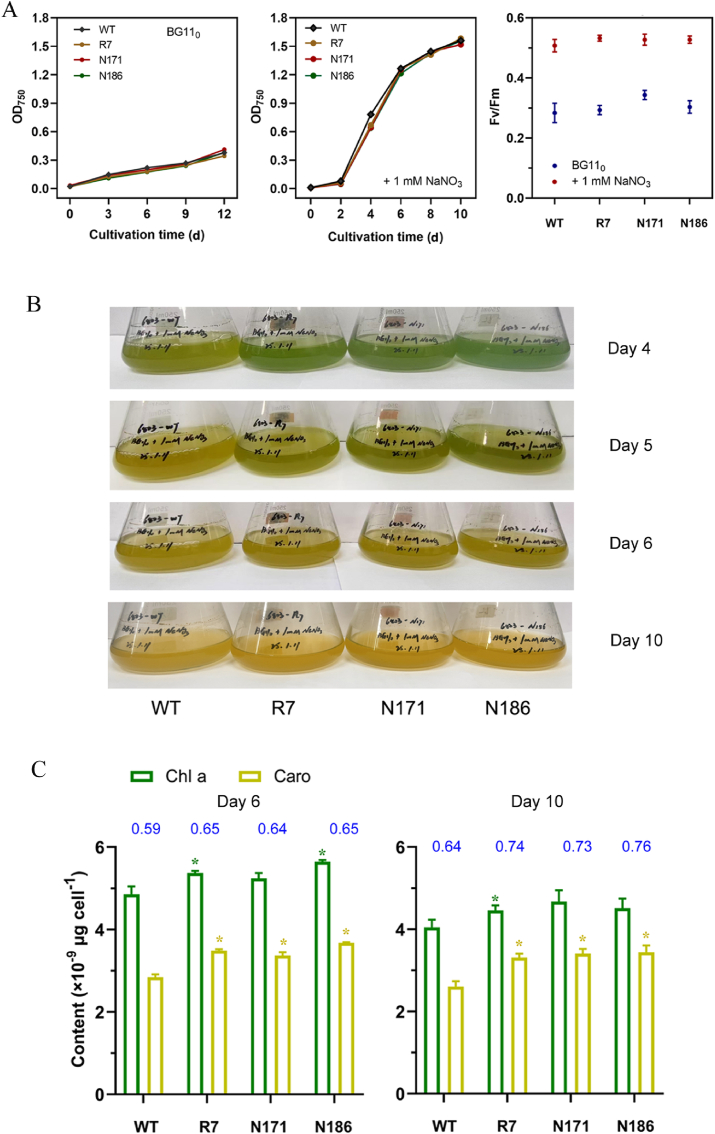
Growth characteristics of transgenic *Synechocystis* sp. PCC 6803 overexpressing the R7, N171, and N186 genes. (A) Growth in BG11_0_ medium, BG11_0_ medium supplemented with 1 mM NaNO_3_, and Photophysiological activity measured as Fv/Fm. (B) Color changes in cultures of transgenic *Synechocystis* sp. PCC 6803. (C) Chlorophyll *a* and carotenoid contents per cell after 6 and 10 days of cultivation. Data are shown as mean ± SD (*n* = 3). ∗Significantly different from the WT (*p* < 0.05, *t*-test). Red values above the columns indicate the average chlorophyll *a*-to-carotenoid ratios. R7, evm.model.contig000115.7; N171, evm.model.contig000029.171. N186, evm.model.contig000024.186.

The transcriptomic analysis revealed that some nitrate assimilation-associated genes were rapidly upregulated under both nitrogen depletion and nitrogen addition (Table S12), suggesting their crucial roles in responding to ambient nitrogen fluctuations. In this study, three candidate genes were selected for heterologous expression in the model cyanobacterium *Synechocystis* sp. PCC 6803 to assess their effects on growth under low-nitrogen conditions (Fig. 6). The selected genes were evm.model.contig000115.7 (named *R7*), evm.model.contig000029.171 (named *N171*), and evm.model.contig000024.186 (named *N186*). R7 and N186 are similar to slr0898 (nirA) and slr0901 (moaA) of *Synechocystis* sp. PCC 6803 respectively [68], while no significant homolog of N171 was identified in this model strain.

When cultivated in BG11_0_ medium, the three transgenic strains exhibited similar slow growth as the wild type (WT) (Fig. 6A). When cultivated in BG11_0_ medium supplemented with 1 mM NaNO_3_, all strains displayed rapid growth after two days of acclimation (Fig. 6A). No obvious differences in growth, as measured by OD_750_, were observed among these strains. However, their photosynthetic activities, in terms of Fv/Fm, were significantly higher under the nitrogen-added condition (Fig. 6A).

Over a cultivation period of up to 10 days, the three transgenic strains exhibited a greener culture color than the WT until day 6, after which they turned yellow (Fig. 6B). Cellular chlorophyll and carotenoid contents, as well as the ratio of carotenoid to chlorophyll, were analyzed on days 6 and 10 (Fig. 6C). The results suggested a decreasing trend in cellular chlorophyll content and an increasing trend in the pigment ratio with prolonged cultivation time, which may explain the changes in culture color. Notably, the cellular chlorophyll contents in the three transgenic strains were significantly higher than that of the WT, implying efficient nitrogen storage in the pigment upon nitrogen recovery. These findings highlight the importance of nitrate assimilation-associated genes in restoring cellular chlorophyll levels, which are crucial for photosynthesis and cell growth.

## 4 Conclusions

The biocrust-derived oleaginous microalga *Vischeria* sp. WL1 is a promising candidate for algal biofuels, owing to its high oil content and excellent adaptability to nitrogen-limiting conditions. In this study, we presented a high-quality assembled genome of *Vischeria* sp. WL1. Comparative genome analysis reveals distinct traits within the genus *Vischeria*. Coupled with transcriptome analyses under nitrogen depletion and repletion conditions, we identified a complete cluster of nitrate assimilation genes in *Vischeria* sp. WL1 that may be responsible for its rapid growth during nitrogen recovery. Nitrogen starvation could regulate the expression levels of genes involved in lipid biosynthesis within 24 h, a response beneficial for enhanced oil accumulation in the subsequent cultivation. Furthermore, heterologous transgenic expression of three nitrogen assimilation-associated genes confirmed their role in restoring cellular chlorophyll level upon nitrogen recovery. Our findings provide insights into the molecular basis of metabolic flux dynamics in oil-producing microalgae under nitrogen depletion and subsequent recovery.

## CRediT authorship contribution statement

**Xiaolan Rao:** Writing – review & editing, Writing – original draft, Formal analysis, Data curation, Conceptualization. **Wensheng Liang:** Investigation, Formal analysis. **Xin Jing:** Investigation, Formal analysis. **Chang Liu:** Investigation, Formal analysis. **Jiahong Li:** Investigation, Formal analysis. **Limei Liu:** Data curation. **Xiang Gao:** Writing – review & editing, Writing – original draft, Funding acquisition, Formal analysis, Data curation, Conceptualization.

## Data availability

The sequencing data and genome assemblies of *Vischeria* sp. WL1 are deposited at CNGB database under accession number PRJCA038099. Other data generated or analyzed during this study are included in this article.

## Declaration of competing interest

The authors declare that they have no known competing financial interests or personal relationships that could have appeared to influence the work reported in this paper.
