# Supplementary material for: Genomic and transcriptomic insights into the molecular responses of a biocrust-derived oleaginous microalga Vischeria sp. WL1 to nitrogen depletion and recovery
Source: Synth Syst Biotechnol. 2025 Jun 14;10(4):1160–71. doi: 10.1016/j.synbio.2025.06.004 (PMC12269273; doi:10.1016/j.synbio.2025.06.004)
Supplement: Multimedia component 1 [file mmc1.docx]

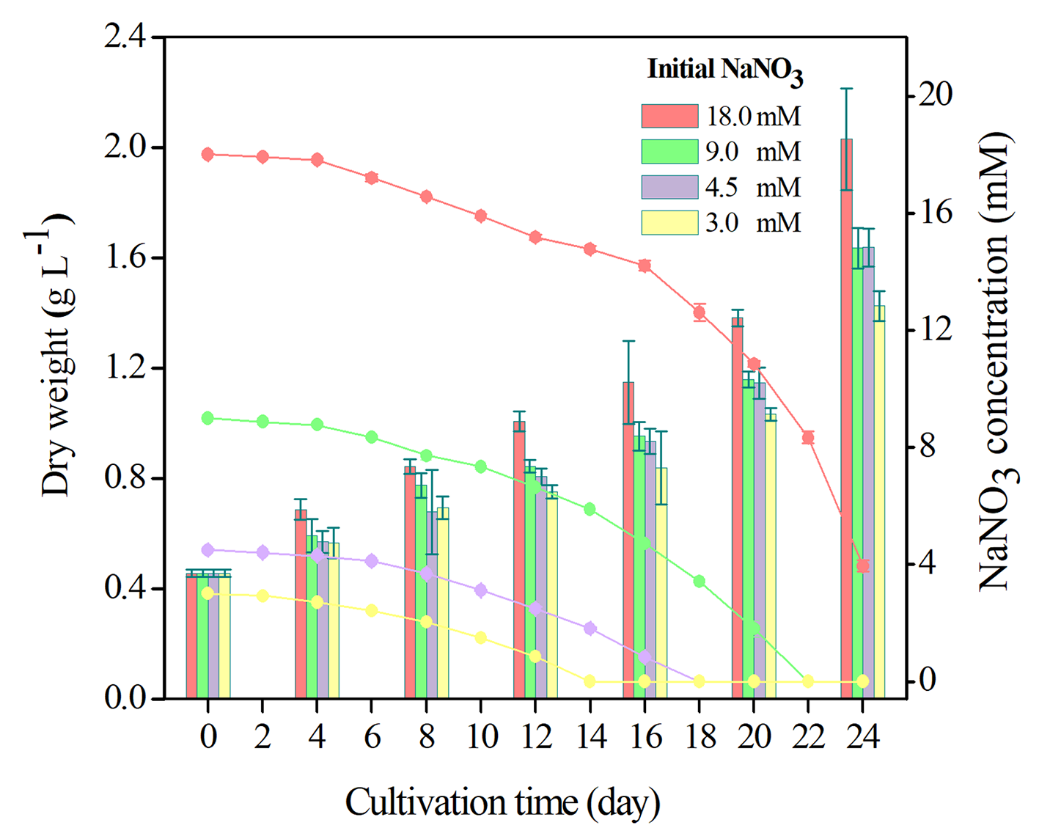


**Figure S1** The biomass change of *Vischeria* sp. WL1 and the nitrogen consumption rate in the media during 24 days of cultivation. Data are shown as mean ± SD (*n* = 3). The BG11_0_ medium was supplemented with different concentrations of NaNO_3_. *Vischeria* sp. WL1 cells were inoculated into 250 mL glass flasks containing 150 mL of medium and subjected to shaking cultivation at 150 rpm for 24 days. Cultivation was performed at 25°C under continuous LED white illumination of 60 μmol photons m⁻² s⁻¹.
