# Supplementary material for: Genomic and transcriptomic insights into the molecular responses of a biocrust-derived oleaginous microalga Vischeria sp. WL1 to nitrogen depletion and recovery
Source: Synth Syst Biotechnol. 2025 Jun 14;10(4):1160–71. doi: 10.1016/j.synbio.2025.06.004 (PMC12269273; doi:10.1016/j.synbio.2025.06.004)
Supplement: Multimedia component 2 [file mmc2.docx]

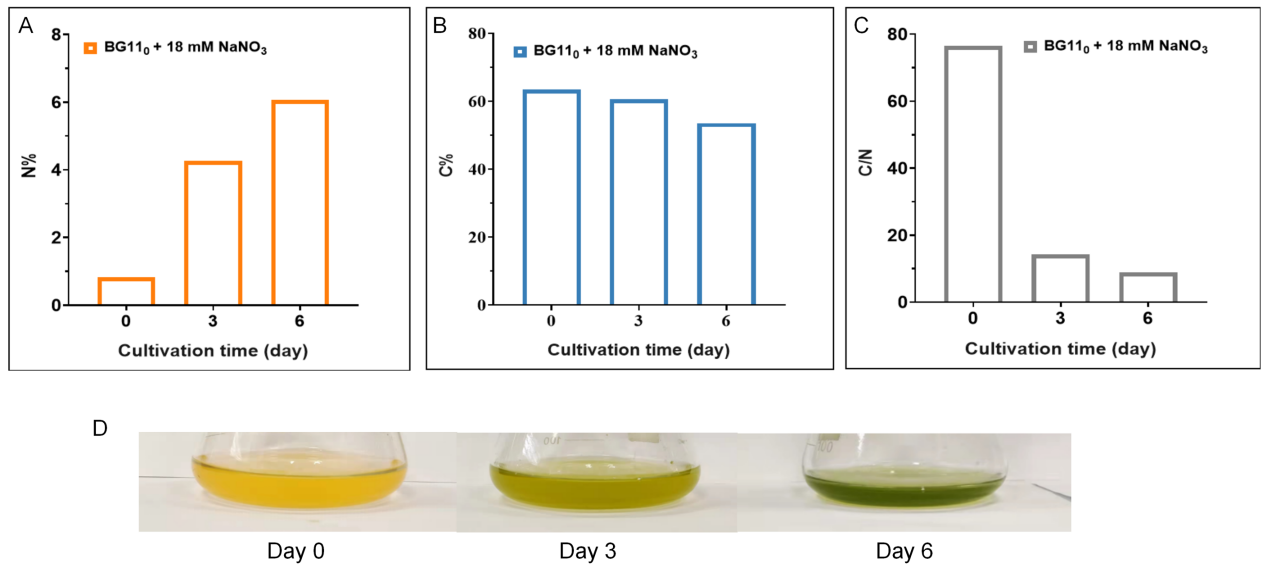


**Figure S2** The changes in N content (A), C content (B), and C/N ratio (C) of *Vischeria* sp. WL1 cultures during 6-day cultivation in BG11_0_ medium supplemented with 18 mM NaNO_3_. D, the color change of *Vischeria* sp. WL1 culture during 6-day cultivation. The starting cells were nitrogen-starved cells that had undergone nitrogen starvation in BG11_0_ medium for 6 months. Cultures were collected by centrifugation and freeze-dried. Approximately 2 mg of each dry sample was subjected to C and N detection using an elemental analyzer (vario EL cube, Elementar, Germany).
