# Supplementary material for: Genomic and transcriptomic insights into the molecular responses of a biocrust-derived oleaginous microalga Vischeria sp. WL1 to nitrogen depletion and recovery
Source: Synth Syst Biotechnol. 2025 Jun 14;10(4):1160–71. doi: 10.1016/j.synbio.2025.06.004 (PMC12269273; doi:10.1016/j.synbio.2025.06.004)
Supplement: Multimedia component 3 [file mmc3.docx]

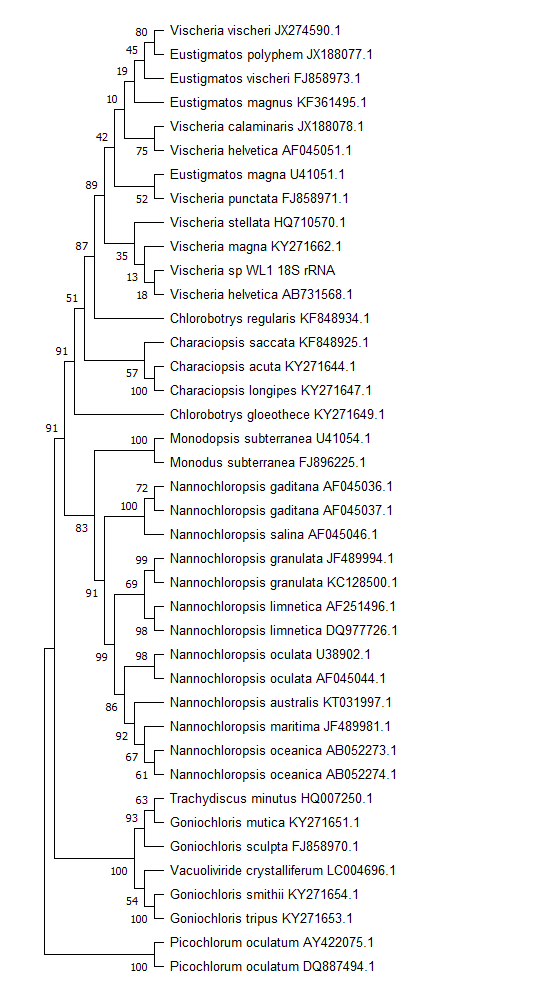


**Figure S3** The phylogenetic tree of 18S rDNA. An 18S rDNA sequence obtained from *Vischeria* sp. WL1 and thirty-nine additional 18S rDNA sequences were aligned. The maximum likelihood (ML) tree with 1,000 bootstrap replicates was constructed.
