# Supplementary material for: Genomic and transcriptomic insights into the molecular responses of a biocrust-derived oleaginous microalga Vischeria sp. WL1 to nitrogen depletion and recovery
Source: Synth Syst Biotechnol. 2025 Jun 14;10(4):1160–71. doi: 10.1016/j.synbio.2025.06.004 (PMC12269273; doi:10.1016/j.synbio.2025.06.004)
Supplement: Multimedia component 4 [file mmc4.docx]

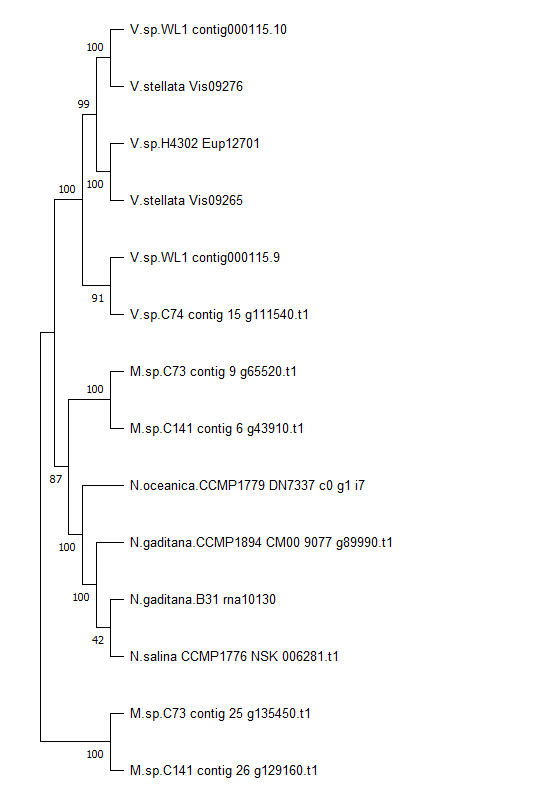


**Figure S4** The phylogenetic tree of NR genes. The NR genes from ten *Eustigmatophyte* genomes were aligned. The maximum likelihood (ML) tree with 1,000 bootstrap replicates was constructed.
