# Supplementary material for: Genomic and transcriptomic insights into the molecular responses of a biocrust-derived oleaginous microalga Vischeria sp. WL1 to nitrogen depletion and recovery
Source: Synth Syst Biotechnol. 2025 Jun 14;10(4):1160–71. doi: 10.1016/j.synbio.2025.06.004 (PMC12269273; doi:10.1016/j.synbio.2025.06.004)
Supplement: Multimedia component 6 [file mmc6.docx]

The synthesized sequence 1 was introduced into the pUC57-simple plasmid to generate the pUC57-6803-frame, which is designed for the expression of a target gene in *Synechocystis* sp. PCC 6803. Three target genes (sequences 2, 3, and 4) were introduced into the pUC57-6803-frame.

**Sequence 1**

(Left 500-bp homology arm + the kanamycin resistance cassette + psbA2 promoter + RBS + four restriction-enzyme cutting sites + B0050 terminator + Right 500-bp homology arm).

**GATATC**

AGGCTTCTCCTCCGGCATTGTGGCCACCCTCACCGCCAGTAAGGTCACCCATCGTAAAATTCGTTCCATCGCCGCCCACTGCAAAAATTCCCTCACCGAAGCGGATTTTCTCAATAACGAAATTTTGATCCATCGCCAAACCACCGCTGATTGGAGCGCGGACTATGGCCAGGTATTGTATCGCCAGGATGGTCTAATCGAAAAGGTTTACACCAGTAATATTGAACCTCTCCACGCTGAATTAGAACATTTTATTCATTGTGTTAGGGGAGGTGATCAACCCTCAGTGGGGGGAGAACAGGCCCTCAAGGCCCTGAAGTTAGCCAGTTTAATTGAAGAAATGGCCCTGGACAGTCAGGAATGGCATGGGGGGGAAGTTGTGACAGAATATCAAGATGCCACCCTGGCCCTCAGTGCGAGTGTTTAAATCAACTTAATTAATGCAATTATTGCGAGTTCAAACTCGATAACTTTGTGAAATATTACTGTTGAATTAATCT

TGTGTCTCAAAATCTCTGATGTTACATTGCACAAGATAAAAATATATCATCATGAACAATAAAACTGTCTGCTTACATAAACAGTAATACAAGGGGTGTTATGAGCCATATTCAACGGGAAACGTCTTGCTCCAGGCCGCGATTAAATTCCAACATGGATGCTGATTTATATGGGTATAAATGGGCTCGCGATAATGTCGGGCAATCAGGTGCGACAATCTATCGATTGTATGGGAAGCCCGATGCGCCAGAGTTGTTTCTGAAACATGGCAAAGGTAGCGTTGCCAATGATGTTACAGATGAGATGGTCAGACTAAACTGGCTGACGGAATTTATGCCTCTTCCGACCATCAAGCATTTTATCCGTACTCCTGATGATGCATGGTTACTCACCACTGCGATCCCAGGGAAAACAGCATTCCAGGTATTAGAAGAATATCCTGATTCAGGTGAAAATATTGTTGATGCGCTGGCAGTGTTCCTGCGCCGGTTGCATTCGATTCCTGTTTGTAATTGTCCTTTTAACAGCGATCGCGTATTTCGTCTCGCTCAGGCGCAATCACGAATGAATAACGGTTTGGTTGATGCGAGTGATTTTGATGACGAGCGTAATGGCTGGCCTGTTGAACAAGTCTGGAAAGAAATGCATAAGCTTTTGCCATTCTCACCGGATTCAGTCGTCACTCATGGTGATTTCTCACTTGATAACCTTATTTTTGACGAGGGGAAATTAATAGGTTGTATTGATGTTGGACGAGTCGGAATCGCAGACCGATACCAGGATCTTGCCATCCTATGGAACTGCCTCGGTGAGTTTTCTCCTTCATTACAGAAACGGCTTTTTCAAAAATATGGTATTGATAATCCTGATATGAATAAATTGCAGTTTCATTTGATGCTCGATGAGTTTTTCTAAAAAATCAAAGGATCTTCTTGAGATCCTTTTTTT

CCGCCAGGTAAACTCTTCTCAACCCCCAAAACGCCCTCTGTTTACCCATGGAAAAAACGACAATTACAAGAAAGTAAAACTTATGTCATCTATAAGCTTCGTGTATATTAACTTCCTGTTACAAAGCTTTACAAAACTCTCATTAATCCTTTAGACTAAGTTTAGTCAGTT

TAGTGGAGGTT

actagt**cccgggtctagactcgag**

**AAAATCAAAGGATCTTCTTGAGATCCTTTTTTT**

CTCAGGGGCATTATCGGAGCAAGCCGATATTCAGGTAAGATGGGGCGGTGCTTTTGTGGGTTGATCCATTAGCTTTAACCCCATTGTCTCCACTGACGGTAATAGTTTTCCCCACTGCGATTAATTGTTTTGTTTGGAGGAGGGACTGCCCCACCGTCAGCCCGCCGCCATAGTTTTGCATTTCCACTGCCATGGGCATCGAACTCCGTAGTTACGTTTACCTGGATAGTCTCCAGTCCCAACATGCAGCCTACATTGGGACGGTGGCCTCCGGCTTTTTGCCGCTACCGGGGGATTGTTCCCTCTGGGTGGAAGTTTCCCCAGGCATTGAAATTAACCGCATTACCGATATTGCTCTTAAGGCAGCGGTGGTGCGGCCGGGGGTATTATTTGTGGAACGGCTCTACGGTTTGTTGGAAATCCATGCCAGTAATCAGGGGGAAGTGCGGGCGGCAGGGCAAGCGATTTTGGCATATATCGGTGCCAAAGCCAGTGACTGC

**GATATC**

**Sequence 2**

**evm.model.contig000115.7 (R7) [mRNA] 1848 bp**

ATGCGCACCGCACTGCCGCTTCTGGCCTTCCTCGGTCTTCATACAGGGTGTTGTGCCTTCGTCGCGCCGACAGGCCCCAGCAGGGCGCATGCGCTAAGCCGTGTTTACTCCACGCTGGAGGAGCGGCCTACGGAGAAGACTGCACGACCGCAACCACGCGCAGATATCAAGGATATAAATCCGCGGGCACTTGAACTGTTTGGGAACCCTTATGAGGCCCCTGCTAAGTTGGGGACCGATTATCTCCCTCCGGAGACACTGGAGAGGGCGAAGAAAGGAAGCTTCGTCGAGAAGATGAAGCTGAAGAAAGACGGTGTGACGCAATGGACGGAGGTGCACGAGCTCGCAACGCTGCTCCGCACAGGGCAGTTCACATGGGAGGAGCTGAACCTGGACGACGCCGATCAGAGGCTCAAGTGGGCAGGACTCTTCCACAGGCGCAAACGCACGCCTGGACGGTTCATGATGAGGATGAAGATCCCCAACGGTATTGTGTCGTCGGATGCGCTGCGATTCATGGCTGAGTCGCTCGTCAAATATGGTGATGATGCGGCTGTCACCATTGACATCACCACCAGACAGAACCTGCAGCTCAGGGGCCTGAAGCTGGAGGACACCAGCGACATCATCGTGGGCCTGGTCGAGCGAGGCCTCGGGTGCTACATGTCGGGCATGGACAACGTGCGCAACATGGTGGGGTCCCCTCTAGCCGGCATCGACCCCCTGGAGCTCTACGATACGCGGCAGCTGTGCGAGGACATCAACAACATGATCACGAACAACAACCGCGGGAATCCGGAGTGGGGCAACCTCCCTCGCAAGTTCAACATTGCGCTTTCGGGATCCAGGGACGACTATGCGCACACGCACATCAACGACATCGGCTTCCAACCCATTGCCAGAGACGGTGTCATGGGCTTCAACGTGGTGGTGGGCGGGTACATGTCGACCAAGCGCGTGGCGTTCAGCGAGGAACTCGATATGTGGGTTCCGCCTTCGGAGGCGGTCAACCTCTGCTACGCCATCCTCAGGCTGTTCCGCGACCACGGCAACCGCAAGGACCGACAGAAGGCGCGTCTGCTTTGGCTCGTGGAGGAGTGGGGCGTCGAGAAGTTCAGGGACGAGGTCATCAAGGAGATGGAGGTCAACCAGGTGTACGGTGGCCACAAGCCGGAAGTGCACCGAGCCGTGGAGCAGCACGGACCGGCATTTGAGAGGAGGGACTACCTCGGCGTGCACAAGCAGAAGCAAGAGGGGTTGAACTGGGTGGGAGTGCACGTGCCCGTAGGAAGGTTCCTGCCCTTCGACGCTTTTGAGATTGCGCGTGTTGCTGACAAGTACTCGAACGGCGAGATCCGCCTGACGGTGGAGCAGAACATCATCTTCCCCAACGTGAAGGACGAGGATGTGGAGTCTCTGCTCTCTGACCCCTTCTTCCAGGGCCGCTTCTCCATCAACCCAGGCCCACTGTCCCGGGCGCTGGTATCGTGCACGGGCGCTCAGTTCTGCGGCGTTGCGCTTGTCGAGACCAAGAACAGGGCCCTCAGGATCGTGGACGAATTGGAGGCCAAGTACCACATCCCTCAAACCGTAAGGATGCACTGGAGCGGGTGCCCCAACCAATGCGGACAGAGCCAGGTCGCGGATATCGGTTTGATGGGGGCGCCCGCGAAGAAGGACGGCAAAGCGGTGGAGGGCGCTGATGTGTTCCTCGGCGGCGCTGTCGGCGAAGTGGCGGAGCTGGGCGAGAAGCTGTACAAGAACGTGGCGTTCGGTGACAACGATGAGGACATCATCAATGTCCTCTCGCAGGTCTTGGTGGAGAAGTATGGGGCTGTGCCAAAGTAG

**Sequence 3**

**evm.model.contig000029.171 (N171) [mRNA] 864 bp**

ATGCGCCGCACCTTGGATGCGGCAAGAGGACTGGCGTCTTCCTTCCGCTCAATGGGAGGCAAGATCAGGCCAAGGGCGACAGGGCTTCGCCCGTTCAGCAGCGTGGAGTTCGACGCGGGCAGTCGGCTCTCAGTACCTTCCAATACAATGGGCGTCTTTGCCAACACTCAACAGCCCTGCATCTCCTATTGGATCGGTCCCAATCTCTACGTGCAGGTGACCAACCGCTGCAATACCACGCCCTTACACGCGACCCGCGGCCCCGGCTTCGCAATGGACACGGACTTCCGCCCTCTGCCTGAGGAATACGAGCCAGCGGCATCGGAGGTGACGCGCAATGTGACGGGCGCTCTGCAGGCGTCCCACTCATCCATGGGGAGAAACAGGCAAGAGGTGGTGTTCGCGGGCTGGGGCGAGCCCACGCTACGCTGGAGCACCGTCCGGGAAGTGGCGGCAAGGATCCGCACGTGCAACCGCAACGTCAGGCTCCGGTTGAGCACGAACGGGCTGGGGAGCTTAGTGATGCAGCGCAACATCGTGCCCGAGGTGCTGTCGTACTTCGACTCGGTGTCCGTGGCGCTCAACACGGCATCTTCGCACCAGTACGACGATATCATGAAGCCCAAGCTGGGCGTGGTGGGCGAGATGGAGGTGAAAGCCATGGAAGTCAAGAGCACCGTGCCGTTCCGCCTGCCCGAGACATACGCCCACGCCATCGTTCGGGAGTTCATCCAGGAGTGCGTGGACGCGGGGGTCCCCACAGAGGTCACAGCAGTCGAGCGGCCCGACGTCAATCTCCACCTGGTCAAGCGCCTCGCCACGTCGATGGGCGCCTCATTCCGCTCGCGCCCCTACTTTGTGTAG

**Sequence 4**

**evm.model.contig000024.186 (N186) [mRNA] 1290 bp**

ATGCGCGGAGTCGTTGGAAAGGTCAAGACAGTGCCGCTTCTTGGGGGTGGCGTTCGTATGCGGGTGAGCCTCCAGTCGACAAGACCACATCGTCGCAGGCTGGAGGACTTCCGTGAGCAGGTTGCTGCTGGGCCCTCGCTCGCAGAGTTCATTGCAGTTGCTCCCAAATGCACGTCTCCGTCACCTCGTCGTGCTGATCAACACGTGCATGCGCCGATTGTGGAGCCTGTTGGGGAGCCTGCCCTCAATTTGAGCCCTCGTGTCAAGGAGCCGCTGGAGCCTGCACGGCATCCTGCATTGCTCGACTCGTTTGGGCGATTTCATAACTACCTGCGGATATCTGTGACGGAGAGGTGTAACCTGCGTTGCACCTACTGTATGCCCGCCGACGGCGTTCCATTGCAGCCATCAGACCGGTTATTGAGCGCTGATGAGATAGTGAGGGTTGCCGAGGTTTTTGTGGGCATGGGGGTCGATAAGATCAGGCTCACGGGCGGCGAGCCGTTGGTTCGCAAGGACCTTGCGTGGCTGACCAGGGCGCTTAGGGGACTGGGTGTGCAGGAAGTAGCGGTCACCACTAATGGTGTCCATCCACCCGCAAGATACGATGAGCTGGTGGACGCAGGCATCACGCAGTTCAACATAAGCCTTGACACGCTGCAGGCGGAAAAGTTTGCACGCATTACACGGAGACCGGGGAAGCTGCTGAAGACCGTGTTGGGGAACATGTATCACTTACTGGAACGGGGGCTGGGTGGGCGAGTCAAAGTGAACAATGTGATCATGCGCGGCGTCAACGACGATGAGCTGGTGGACTTCGTGATGTTGACGCGAGATCGGCCTTTGGATGTTAGATTCATAGAGTGGATGCCGTTTGACAGCAATGGGTGGAATGACAGGACCTTCTTCTCCTATAAAGAGATTATAGCGCGCATCACGGATGCTTTTCCGCGGTTCGCGCGGGATGTGGATGGGCCAAATGATACGACGAAGTGGTATAGAGCTGCGGACCACATTGGGCGCGTGGGGTTTATATCGTCGATGAGCCAACACTTCTGTGGCGATTGCAATCGCTTGAGGATCACTGCGGACGGGAAGCTCAAGGTCTGCCTGTTTGGCGAGGAGGGGCTGAGCTTGCGTGATGCTGTTAGAGGTGGGAAGAGCATCGACGAATTACAGGAGCTAATAGCGAGCGCCGTGGGACGCAAGAAGGCTGTACTTGGTGGTCGAGGTGACATGTATGGTCTAGCCAAGTCGAAGAACAGACCAATGATCTTGATTGGCGGATGA
